# Advanced drug delivery and PBPK modelling as translational tools for precision therapies in juvenile idiopathic arthritis

**DOI:** 10.3389/fphar.2026.1917187

**Published:** 2026-10-14

**Authors:** Marina Cuquerella-Gilabert, Cristina Martin-Sabroso, Javier Zarzoso-Foj, Juan Aparicio-Blanco, Alejandro Balsa, Ana Isabel Torres-Suárez, Víctor Mangas-Sanjuán, Carlos Rodríguez-Nogales

**Affiliations:** 1 Department of Pharmacy and Pharmaceutical Technology and Parasitology, University of Valencia, Valencia, Spain; 2 Interuniversity Research Institute for Molecular Recognition and Technological Development, Polytechnic University of Valencia – University of Valencia, Valencia, Spain; 3 Department of Pharmaceutics and Food Technology, Faculty of Pharmacy, Complutense University of Madrid, Madrid, Spain; 4 Institute of Industrial Pharmacy, Complutense University of Madrid, Madrid, Spain; 5 Rheumatology Unit, La Paz University Hospital, Madrid, Spain

**Keywords:** arthritis, controlled release, DMARDs, drug delivery systems, juvenile idiopathic arthritis, long-acting formulations, PBPK, TMDD

## Abstract

Patients with persistent forms of juvenile idiopathic arthritis (JIA) often report limited therapeutic benefit from currently available treatments. Despite substantial advances in the management of rheumatoid arthritis (RA) and related disorders over recent decades, treatment strategies for JIA remain insufficiently defined. The application of advanced drug delivery systems holds promise in addressing these limitations by enabling controlled and sustained drug release over extended periods. Other integrative approaches such as novel physiologically based pharmacokinetic (PBPK) modelling have demonstrated to be a critical tool in optimizing pharmacotherapy in pediatric populations, particularly by improving dose selection and predicting drug disposition. This review summarizes current disease-modifying antirheumatic drugs (DMARDs) for JIA management and critically examines the current, still largely prospective, integration of emerging drug delivery systems with PBPK modelling, highlighting existing translational examples alongside key opportunities and challenges for precision therapies in this field.

## 1 Introduction

Juvenile idiopathic arthritis (JIA) is the most prevalent chronic inflammatory rheumatic disease in children, affecting approximately three million children and young adults worldwide (Huang H. Y. R. et al., 2024). In the most severe or difficult-to-treat cases, such as polyarticular JIA, the disease impacts all aspects of life, extending beyond physical disability and frequently persisting into adulthood (Pagnini et al., 2016). Furthermore, evidence indicates a progressive decline as patients age into adolescence, reflecting a broader psychological burden. Some studies highlighted significant psychosocial challenges, with more than half of adolescents expressing experiences of isolation and perceived social exclusion (LeLeiko et al., 2013; McKeever and Kelly, 2015). In such cases, the limited benefit from therapies correlates with poor adherence and treatment fatigue. A 2023 study reported that at least one-fourth of pediatric patients with rheumatic diseases, particularly those with polyarticular JIA and enthesitis-related arthritis, exhibited poor medication adherence (Manatpreeprem et al., 2023).

Despite significant advances over recent decades in the management of rheumatoid arthritis (RA) and related diseases, treatment protocols for JIA remain insufficiently defined. Current therapeutic approaches often require parenteral administration, which can lead to undesirable and, in some cases, serious adverse effects, particularly in the pediatric population. This challenge is evident in refractory cases, where current therapeutic options yield suboptimal outcomes. Although still underexplored in pediatric populations, the implementation of advanced drug delivery systems offers promising potential to overcome these limitations by enabling controlled and sustained drug release over extended periods. In parallel, integrative approaches such as physiologically based pharmacokinetic (PBPK) modelling have demonstrated considerable utility in optimizing pharmacotherapy in pediatric patients by improving dose selection and predicting drug behavior. This review aims to describe the prospective role of these novel therapeutic approaches in JIA, highlighting current research while also considering relevant clinical perspectives, opportunities and challenges. Articles and clinical trials addressing the aforementioned topics and published mostly within the previous 10 years were considered for inclusion in this review. Literature search was performed using MEDLINE and ClinicalTrials.gov database (U.S. National Library of Medicine).

## 2 Understanding juvenile idiopathic arthritis

The pathophysiology of most JIA categories remains unknown, thereby limiting early intervention, optimal disease management, and the development of personalized therapies. This heterogeneous condition is defined as arthritis persisting for more than 6 weeks in patients under 16 years of age. There are seven recognized subtypes (systemic arthritis, oligoarthritis, rheumatoid factor (RF)-negative or RF-positive polyarthritis, psoriatic arthritis, enthesitis-related arthritis, and undifferentiated arthritis) classified according to clinical presentation during the first 6 months of disease onset (Ravelli and Martini, 2007). These subtypes differ not only in clinical manifestations and prognosis, but also in their underlying immunopathological mechanisms and therapeutic responses (Murakami et al., 2012). Beyond synovial inflammation and progressive joint destruction, JIA may involve extra-articular tissues including the eyes (uveitis in oligoarthritis), skin, gastrointestinal tract, and internal organs, particularly in systemic JIA (Zaripova et al., 2021; Patel et al., 2026) (Figure 1).

**FIGURE 1:**
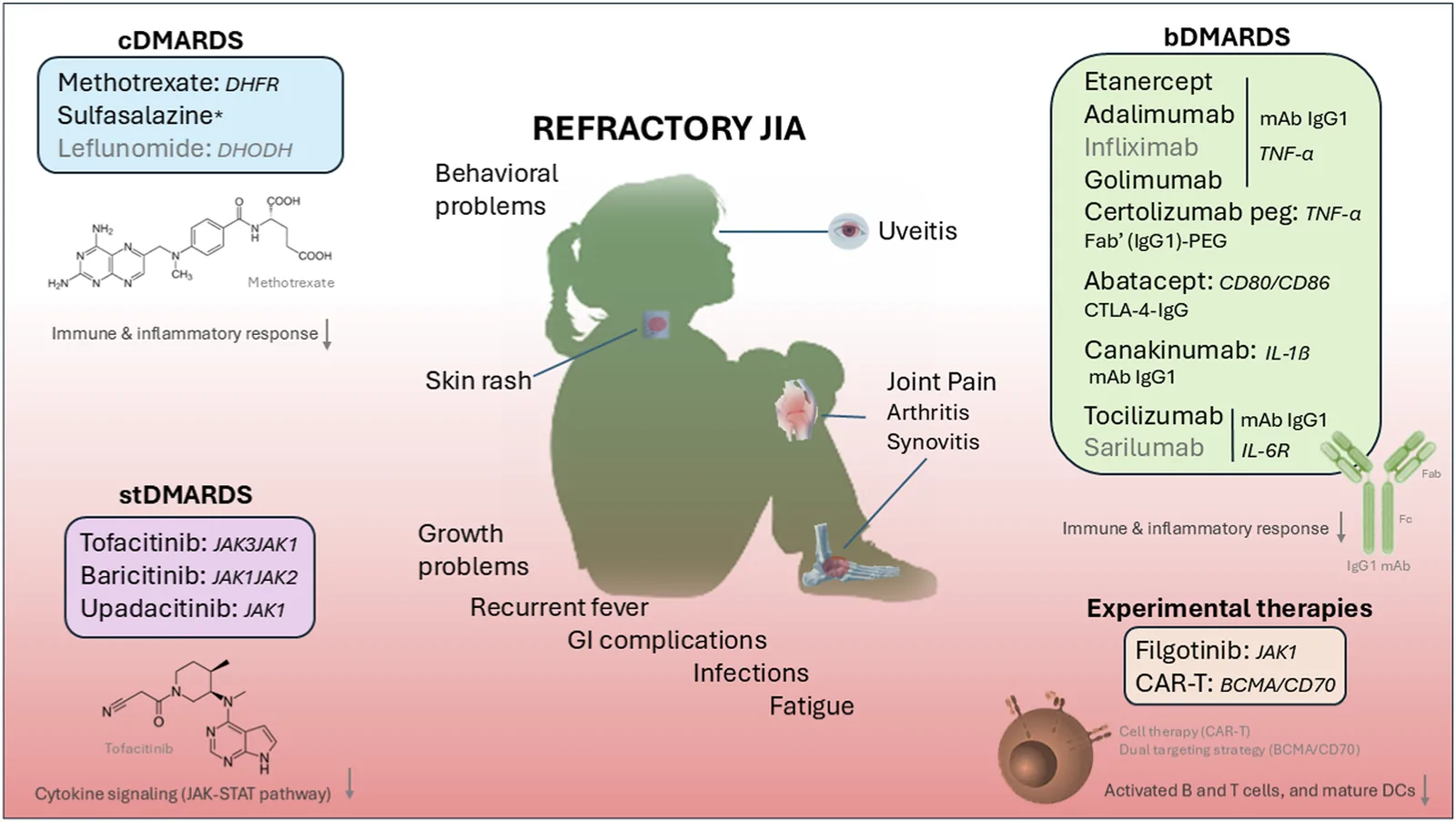
Refractory JIA symptoms and current therapeutic arsenal (cDMARDS, bDMARDS, stDMARDs and experimental candidates). Therapies in grey represent off-label indications. *Target remains unknown.

At the molecular level, JIA is characterized by a dysregulated immune response, including aberrant activation of T and B lymphocytes, macrophages, dendritic cells, neutrophils, and natural killer (NK) cells, and excessive production of proinflammatory cytokines such as tumor necrosis factor (TNF)-α, interleukin (IL)-1, IL-6, and IL-17 (Pagnini et al., 2016). These mediators maintain synovial hyperplasia, cartilage degradation, and bone erosion. However, significant differences in the biology and pathogenesis of the disease lead to variability in responses to medical treatment (Peitz and Horneff, 2015). Emerging therapeutic targets include IL-17/IL-23 pathways, the granulocyte–macrophage colony-stimulating factors, and JAK cascade (Kilic Konte et al., 2026).

## 3 Current therapeutic landscape

Therapeutic management of JIA is increasingly guided by early, treat-to-target disease control, aiming for remission or low disease activity while preventing joint damage, extra-articular morbidity, growth impairment, and prolonged glucocorticoid exposure. As shown in Figure 1, current protocols primarily rely on conventional disease-modifying antirheumatic drugs (cDMARDs), such as methotrexate (MTX), sulfasalazine, and leflunomide. However, their effectiveness is frequently compromised by toxicity, complex dosing regimens, and poor adherence. MTX, for instance, remains the first-line therapy for non-systemic and polyarticular-course JIA, yet approximately 50% of pediatric patients experience adverse effects such as gastrointestinal symptoms, hepatotoxicity, and behavioral disturbances, leading to treatment discontinuation in nearly 35% of cases (Giannini et al., 1993; Beukelman et al., 2012; Barral Mena et al., 2020). Additionally, MTX is a known teratogen, necessitating contraceptive counselling for sexually active adolescent females. Nonetheless, relapses occur in 30%–50% of patients. Leflunomide is generally considered to be less effective and is prescribed only for off-label use. Sulfasalazine is reserved for selected phenotypes and avoided in systemic JIA since severe adverse events, including macrophage activation syndrome, have been described (Silverman et al., 2005; Van Rossum et al., 2007).

Patients with difficult-to-treat JIA, defined by failure to achieve inactive disease, steroid-sparing control, or acceptable functional status, require long-term immunosuppressive therapy with biologic disease-modifying antirheumatic drugs (bDMARDs) (Pagnini et al., 2016; Nijhuis et al., 2023; Yalamanchili et al., 2025). TNF antibodies are the most used, particularly etanercept and adalimumab, with infliximab (off-label), golimumab and, more recently, certolizumab pegol. Certolizumab pegol is a PEGylated, humanized anti-TNFα Fab’ fragment without the Fc region and is indicated by the FDA for active polyarticular JIA in patients ≥2 years, with pediatric efficacy supported by pharmacokinetic exposure and extrapolation from RA. As depicted in Figure 1, alternative targets include IL-6R (tocilizumab and off-label sarilumab) or IL-1 (canakinumab). The first is mainly used for systemic JIA, whereas the second is reserved for refractory cases. Nevertheless, only 50% of patients treated with bDMARDs achieve remission within 5 years. These limitations have prompted the exploration of alternative therapeutic strategies, including targeted synthetic DMARDs (stDMARDs). Most of these agents belong to the Janus kinase (JAK) inhibitor family, which modulate inflammatory and autoimmune pathways by inhibiting cytokine-mediated signaling. In this regard, clinical trials evaluating JAK inhibitors have demonstrated efficacy over several clinical outcomes. Tofacitinib has been reported to reduce flare risk in polyarticular-course JIA, whereas baricitinib, and upadacitinib have shown rapid improvements in disease activity, pain, function, and health-related quality of life (Ruperto et al., 2017; Ramanan et al., 2023; Brunner et al., 2025). In 2024, the FDA approved Rinvoq® (upadacitinib extended-release tablets) and Rinvoq® LQ (1 mg/mL oral solution), a new pediatric immediate-release (IR) formulation, for the treatment of active polyarticular JIA and active psoriatic arthritis in patients of 2 years of age and older (He et al., 2026). Nevertheless, long-term pediatric safety remains a key limitation, particularly regarding infections, herpes zoster, and cytopenias (Ruperto et al., 2021). In 2026, filgotinib entered Phase III clinical trials for the treatment of patients with polyarticular or systemic JIA (NCT07553182).

Beyond established therapeutic pathways, a recent Phase I clinical trial has been initiated in patients with polyarticular JIA to evaluate a dual-targeted BCMA/CD70 chimeric antigen receptor T-cell (CAR-T) therapy (NCT07184450), highlighting the emergence of advanced cell-based immunotherapies in the treatment of refractory pediatric autoimmune diseases (Ciurtin et al., 2025).

## 4 Advanced drug delivery systems

Advanced drug delivery systems including nanotherapeutics have been developed in the last decades to protect therapeutic agents and biomolecules (e.g., small molecules, peptides, and nucleic acids) from degradation in biological environments while enhancing their therapeutic index (Bukke et al., 2026; Wang et al., 2026). These systems encompass a wide range of platforms, including nanoparticles, microparticles, hydrogels, and microneedles, among others. As depicted in Figure 2A, controlled drug release can help improve treatment adherence by extending dosing intervals (prolonged-release systems), which is particularly relevant for chronic diseases such as JIA. They can also modulate inherent drug pharmacokinetic and biodistribution, potentially reducing off-target effects, which are particularly relevant for drug substances associated with high systemic toxicity and low half-life (Bruno et al., 2022; Larrañeta and Domínguez-Robles, 2025).

**FIGURE 2:**
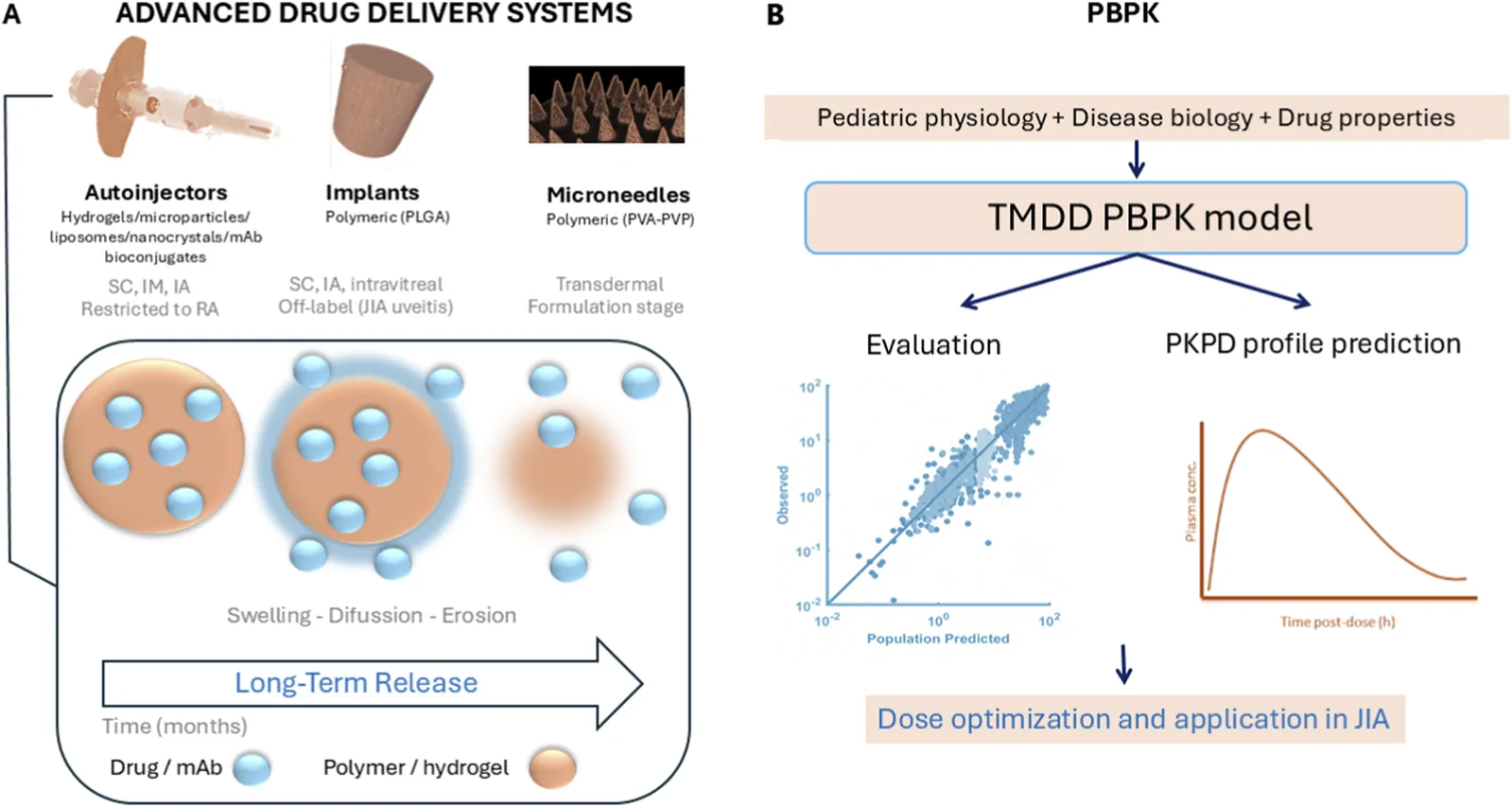
**(A)** Advanced drug delivery systems, release mechanisms and micro-structure of a controlled release delivery. SC, subcutaneous; IM, intramuscular; IA, intra-articular. **(B)** TMDD PBPK schematic workflow. Within the model-informed drug development (MIDD), PBPK modelling provides a particularly valuable framework for JIA because it can connect pediatric physiology, disease biology, drug properties, target engagement and clinical outcomes within a mathematical framework.

Advances in precision therapy for JIA patients, addressing, for example, pediatric dosing requirements through dose adjustment, have been explored *via* the oral route by combining hot-melt extrusion and 3D printing. In this regard, Huang et al. were able to manufacture indomethacin printlets by fused deposition modelling using drug-loaded filaments with distinct release rates, thereby enabling once- or multiple-daily administration according to individual needs and requirements (Huang et al., 2024b). Research on novel drug delivery systems for JIA remains extremely limited and is dismal in the context of novel bDMARDs and stDMARDs. Some studies have reported the off-label use of marketed prolonged release dosage forms to treat symptoms of JIA. This is the case of the biodegradable dexamethasone intravitreal implant Ozurdex® used in patients with recalcitrant JIA-related uveitis to restore anterior inflammation and macular edema (Pichi et al., 2017). The FDA-approved biocompatible polymer poly(lactic-co-glycolic acid) (PLGA) based implant is compatible with a multidose regimen interval of 6 months following local delivery. Other studies have reported the design and development of delivery systems conceived for RA and JIA. This is the case of hydrogel-forming microneedles for transdermal MTX delivery following minimally invasive administration (Tekko et al., 2020). Their pharmaceutical development based on drug diffusion through a swollen hydrogel-forming matrix was intended to pose an alternative to MTX oral administration, which is often associated with side effects and non-linear pharmacokinetics phenomena. However, their effects are yet to be tested in a preclinical model of JIA disease.

Despite the remarkable success of biologics, controlled release formulations for mAbs remain an unmet clinical need for JIA. Alternatively, targeted anti-TNF mAb bioconjugates have been developed for RA treatment through the combination of a peptide that selectively binds denatured collagen present in inflamed joints (Mo et al., 2026). For RA, some specific drug delivery systems have also reached clinical trials. This includes the first-in-class phase II Nanocort trial (NCT00241982, completed), which demonstrated superior response rates of intravenous (IV) pegylated liposomal prednisolone compared with standard intramuscular (IM) methylprednisolone (Zha et al., 2026). Other phase II trials for RA are currently recruiting patients. This is the case of the NCT06565273 trial, which evaluates the efficacy of folate-based liposomes encapsulating MTX following IV or subcutaneous (SC) administration.

## 5 PBPK modelling

Unlike empirical PK models, PBPK models are structured upon a mechanism-driven paradigm, representing the body as a network of physiological compartments (e.g., liver, kidney, brain) interconnected by blood circulation, allowing with a greater degree of certainty prediction not only of systemic exposure but also tissue-site concentrations that can subsequently drive PK/PD models (Jones and Rowland-Yeo, 2013; Santos et al., 2025). PBPK models can support a Predict–Learn–Confirm workflow: initial models built from physicochemical, *in vitro*, preclinical and sparse clinical data can be used to predict pediatric exposure and trial scenarios; emerging PK, biomarker and efficacy data can then refine and validate the model (Jones and Rowland-Yeo, 2013; Suri et al., 2015). Such a mechanistic foundation provides PBPK modeling with remarkable extrapolation capability (Li et al., 2025). This is critical in JIA, where response to TNFα-, IL-6R-, IL-1- or JAK/STAT-directed therapies depends on drug exposure in inflamed synovial and immune compartments, target expression and turnover, and the age-, weight-, growth- and inflammation-related variability of children. The increasing emphasis on treat-to-target and personalized approaches in JIA, together with the recognition of its biological heterogeneity, and the potential of biomarkers or artificial intelligence-supported tools to guide the selection between early aggressive and step-up strategies, highlights the growing relevance of mechanistic prediction approaches (Melki and Frémond, 2023; Wong et al., 2025).

For classical biologics and especially next-generation candidates proposed for JIA, PBPK models should extend beyond classical ADME and explicitly incorporate target-mediated drug disposition (TMDD). This requires describing non-specific distribution and elimination, including extravasation into interstitial and synovial spaces, lymphatic return, pinocytosis, proteolytic catabolism and FcRn- or albumin-related recycling, together with specific processes such as association/dissociation with TNFα or IL-6R, formation of drug-target complexes, receptor or cytokine turnover, complex internalization, and target-dependent complex degradation or clearance. This level of mechanistic detail is essential because PBPK-PD models show that target abundance and internalization rate of drug-target complexes can be major determinants of distribution (Wang et al., 2025). Translating these principles to JIA would allow PBPK/TMDD models to be coupled with clinically relevant PD outcomes (Figure 2B). Virtual JIA cohorts could then be used to simulate alternative dosing intervals, body-weight strata, disease subtypes, adherence patterns and biomarker-defined endotypes, helping to identify exposure thresholds for remission, anticipate loss of response driven by high target burden or accelerated complex clearance, and compare full IgGs, Fab’ fragments and nanobodies before pediatric trials.

In this regard, and despite the limited number of models developed specifically for JIA (Table 1), a PBPK model has been recently developed to establish optimal dosing regimens of adalimumab in pediatric patients with JIA, which demonstrates the potential of this type of strategies to integrate data from different sources and efficiently optimize the decision-making process during drug development (Liu et al., 2025). More recently, a PBPK/TMDD model for tocilizumab in systemic JIA was developed to support dose-escalation strategies and evaluate vaccination timing from a safety perspective (Yang et al., 2026), highlighting the emerging, albeit still limited, application of this modelling approach to JIA-specific biologics. Beyond JIA, PBPK modelling has also been applied to pediatric biologics such as infliximab in inflammatory bowel disease, both in comparison with allometric scaling approaches (Malik and Edginton, 2019) and within a population PBPK framework (Chang et al., 2022), supporting the broader feasibility of this approach for large-molecule immunomodulators in children. PBPK modelling is used throughout the drug discovery, during hit and lead generation, pre-clinical development and in translational studies (Suri et al., 2015; Perry et al., 2020). Therefore, PBPK models benefit drug development by reducing animal studies and costs, guiding experimental design, testing hypotheses, and understanding the mechanisms of underlying processes, representing a central translational tool to rationalize dose selection in JIA patients.

**TABLE 1:** Existing PK/popPK/PBPK/TMDD models relevant to JIA or evaluated using JIA-inclusive pediatric data.

Model type	Drug	References	Key features relevant to JIA
popPK	Canakinumab	Sun et al., 2016	IL-1β binding kinetics informing dose recommendations in systemic JIA
popPK	Abatacept	Gandhi et al., 2021	Model-based selection of subcutaneous dose in polyarticular JIA
popPK	Tofacitinib	Chang et al., 2024	PK characterization in children and adolescents with JIA
popPK (allometric)	Baricitinib	Decker et al., 2024	Allometric-scaling population model in JIA
popPK	Emapalumab	Brossard and Laveille, 2024	IFNγ-dependent clearance in systemic JIA/macrophage activation syndrome
popPK	Ustekinumab	Lam et al., 2025	Real-world PK (U-POPS) in juvenile psoriatic arthritis
PBPK/TMDD	Adalimumab	Liu et al., 2025	Predicts exposure and dosage regimens in pediatric JIA
PBPK/TMDD	Tocilizumab	Yang et al., 2026	Dose-escalation and live-vaccine-timing safety model in pediatric systemic JIA
PBPK (non-JIA-specific evaluation)	Infliximab	Malik and Edginton, 2019; Chang et al., 2022	PBPK vs. allometric scaling for large-molecule pediatric PK, evaluated using inflammatory bowel disease and JIA data

Establishing an integrated workflow, in which PBPK/TMDD models (Figure 2B) integrate formulation-derived drug release kinetics and inform formulation design objectives, remain a significant methodological challenge in JIA. Within this bidirectional framework, the *in vitro* drug release and absorption kinetics characterized during formulation development (e.g., the burst and sustained-release profiles of LAIs, Figure 2A) can be incorporated as mechanistic input functions into the absorption component of a PBPK model, rather than being treated as separate and purely descriptive attributes. The resulting PBPK/TMDD model establishes a quantitative link between local and systemic drug exposure and target engagement, thereby enabling the *in silico* evaluation of alternative release profiles, dosing intervals, and formulation strategies prior to clinical testing. In turn, model-informed predictions can facilitate clinical translation by identifying promising dosing regimens, supporting trial design, and flagging formulations that are unlikely to maintain adequate target occupancy throughout the dosing interval.

## 6 Discussion

SC and IM drug delivery has the potential to reshape treatment paradigms for systemic conditions such as RA and JIA. This transition, facilitated by wearable devices and auto-injectors/injection pens (Figure 2A), is associated with improved tolerability and convenience compared to IV administration and, to a lesser extent, oral administration (Bianchi et al., 2016). The MetoJect® pen is an auto-injector pre-filled with MTX, currently indicated for active and severe JIA. Although it ensures complete absorption, it does not inherently provide controlled-release properties. Long-acting injectable (LAI) formulations have emerged as a transformative therapeutic platform to address key challenges in chronic disease management. Evidence from other therapeutic areas, such as psychiatry, indicates that LAIs significantly reduce relapse rates and improve adherence among pediatric populations struggling with daily oral regimens, suggesting clear benefits for vulnerable groups (Levy et al., 2025). However, it is important to note that LAI use in children and adolescents (<18 years) remains largely off-label, underscoring the urgent need for age-specific research.

Technological advancement faces additional challenges when applied to novel small molecules such as stDMARDs. These compounds are commonly classified as Biopharmaceutics Classification System (BCS) class II or IV drugs (Amidon et al., 1995). In particular, their poor aqueous solubility not only severely limits oral bioavailability but also poses important hindrances for the development of parenteral formulations (Singh et al., 2024). In this context, nanocrystals and nanosuspensions represent promising and realistic LAI candidates, as they enable high drug loading and controlled release profiles (Rossier et al., 2024). Some of these systems have already been evaluated in preclinical RA models and are progressively gaining FDA approval for parenteral administration (Morici et al., 2025). Furthermore, embedding drugs or drug nanocrystals within hydrophobic, biocompatible, and biodegradable microparticles has been shown to prolong drug release for several months while increasing local residence time (Rodríguez-Nogales et al., 2023). These approaches slow clearance from the injection site and promote depot formation. However, clinical translation remains challenging. Maintaining batch-to-batch consistency and critical quality attributes during scale-up, together with the need for robust physicochemical and biological characterization, can hinder the transition from laboratory-scale production to GMP-compliant manufacturing. Moreover, regulatory requirements for complex delivery systems and the limited predictability of preclinical models may further delay clinical development. Early consideration of these aspects and closer collaboration between academia, industry, and regulatory authorities are therefore essential to facilitate successful translation into the clinic.

The high molecular weight and complex structural architecture of mAbs pose significant challenges for their incorporation into controlled-release systems (Singh et al., 2024). Polymer-based delivery platforms, particularly those based on PLGA nanoparticles or microparticles, have consistently failed to achieve sustained release beyond a few weeks. This limitation is likely due to low loading efficiency, pronounced initial burst release, and protein instability. Implantable devices, even under optimal conditions, release only approximately 50% of their payload within 30 days, which remains insufficient for achieving meaningful clinical impact (Waterkotte et al., 2022; Chang et al., 2025). The nano- and micro-encapsulation of next-generation antibody-based therapeutics, including nanobodies, represents a promising strategy to overcome these limitations. For instance, vobarilizumab (ALX-0061) is an investigational bispecific nanobody targeting soluble IL-6 receptor (sIL-6R) and albumin, designed to combine potent immunomodulatory activity with improved pharmacokinetic properties. Although it reached Phase II clinical trials, its development was subsequently discontinued in RA and systemic lupus erythematosus (Jin et al., 2023; Alexander and Leong, 2024).

One of the major shortcomings in early-stage development has been the inadequate evaluation of the PK profile of LAIs and its relationship with disease-specific biomarkers and PD outcomes. SC administration of LAIs frequently exhibits complex absorption kinetics influenced by lymphatic uptake, local tissue binding, and formulation-dependent release mechanisms. In this setting, PBPK models enable mechanistic characterization of processes such as partitioning between blood capillary and lymphatic uptake at the SC injection site (Offman et al., 2016), saturation of FcRn-mediated recycling pathways (Stader et al., 2024), and the impact of molecular size (Li et al., 2021), particularly for large molecules such as bDMARDs compared to small-molecule drugs, including stDMARDs. A key aspect for optimizing precision therapies with bDMARDs in patients with JIA is the longitudinal characterization of cytokines such as IL-6 and TNF-α, as drug–target complex formation is directly linked to disease progression and therapeutic response (Walters et al., 2016; Morozova et al., 2024). Several popPK models have been published to support dose recommendations of canakinumab (Sun et al., 2016), abatacept (Gandhi et al., 2021), tofacitinib (Chang et al., 2024), baricitinib (Decker et al., 2024), emapalumab (Brossard and Laveille, 2024) and ustekinumab (Lam et al., 2025) in JIA patients. Although they present IL-1β binding kinetics (canakinumab) and IFNγ-dependent clearance in macrophage activation syndrome (emapalumab) in systemic JIA, these models lack to capture tissue exposure, target occupancy, and synovial physiology, limiting their extrapolation ability. In this context, TMDD modeling describes the phenomenon whereby high-affinity drug-target interactions significantly influence the PK properties of a compound and is considered the primary mechanism underlying nonlinear distribution and elimination kinetics (Levy, 1994; Mager and Jusko, 2001). TMDD-related clearance may contribute substantially to overall drug elimination (Mager, 2006), particularly when drug molar concentrations are low relative to target abundance, a situation commonly observed with bDMARDs.

In the context of JIA, PBPK modeling is emerging as a critical quantitative tool for pediatric extrapolation, allowing the integration of age-dependent physiological, biochemical, and anatomical changes to predict drug pharmacokinetics across different stages of childhood (Sule et al., 2025). Unlike empirical allometric scaling, PBPK models mechanistically account for developmental trajectories in organ growth, tissue perfusion, plasma protein composition, and the ontogeny of drug-metabolizing enzymes and transporters, thereby improving the accuracy of dose selection in infants and young children (Zhao et al., 2011; Barrett et al., 2012). By integrating *in vitro* ADME data with system-specific anatomical and physiological parameters, PBPK models support the identification of safe and pharmacologically relevant starting doses, optimization of dose-escalation strategies, and anticipation of inter-individual variability in drug exposure prior to clinical studies. Regulatory agencies, including the European Medicines Agency (EMA; EMA/CHMP/458101/2016) and the U.S. Food and Drug Administration (FDA; FDA-2016-D-3969), increasingly recognize PBPK modelling as a key component of pediatric drug development, particularly in scenarios where clinical data are limited or ethically challenging to obtain (Leong et al., 2012; Johnson et al., 2022). These models are especially valuable for drugs with complex absorption processes, such as advanced drug delivery systems (LAIs), and for compounds exhibiting nonlinear pharmacokinetics driven by target-mediated clearance, as is the case for many bDMARDs (Shebley et al., 2018).

Despite their translational value, PBPK/TMDD models for JIA face several limitations. Pediatric-specific system data remain scarce: synovial fluid volume, local lymphatic flow, and target (cytokine/receptor) turnover in inflamed joints are largely extrapolated from adult or preclinical sources, introducing structural uncertainty. TMDD parameters (association/dissociation rates, complex internalization, target synthesis/degradation) are difficult to identify from the sparse pediatric sampling schemes typical of JIA trials, which can lead to parameter non-identifiability and over-parameterization. As discussed above, current popPK models for canakinumab, abatacept, tofacitinib, baricitinib, emapalumab and ustekinumab in JIA do not capture tissue exposure, target occupancy or synovial physiology, limiting their extrapolation ability. Disease activity itself is time-varying and can modulate target expression and vascular permeability, yet is rarely incorporated as a dynamic covariate. Immunogenicity, which can accelerate clearance of bDMARDs, is also seldom integrated into current PBPK/TMDD frameworks. The formation of anti-drug antibodies can increase drug clearance, reduce circulating drug concentrations and, in some cases, neutralize biological activity, thereby contributing to treatment failure and hypersensitivity reactions. These limitations underscore the need for rigorous model validation before models are used to support dosing decisions. Robust validation, including internal, external, and prospective qualification procedures aligned with regulatory guidance, is essential to ensure the credibility of PBPK/TMDD models and to prevent overconfident dosing predictions in pediatric JIA populations with limited clinical data.

## 7 Conclusion

This article proposes the implementation of advanced drug delivery systems such as LAIs to enable controlled release of novel DMARDs over extended periods. There is also a need for a mechanistic understanding of the PK/PD processes that take place after the administration of bDMARDS and stDMARDS for JIA to efficiently design dosing regimens that maximize treatment efficacy. Coupling the advantages of these two translational tools will be fundamental to developing effectively precision therapies in pediatric patients with JIA.
